# Anti‐obesity and hypolipidemic effect of water extract from *Pleurotus citrinopileatus* in C57BL/6J mice

**DOI:** 10.1002/fsn3.962

**Published:** 2019-03-05

**Authors:** Yao Sheng, Changhui Zhao, Shujuan Zheng, Xiaohong Mei, Kunlun Huang, Guoyi Wang, Xiaoyun He

**Affiliations:** ^1^ Beijing Advanced Innovation Center for Food Nutrition and Human Health College of Food Science and Nutritional Engineering China Agricultural University Beijing China; ^2^ Department of Food Quality and Safety College of Food Science and Engineering Jilin University Changchun China; ^3^ Logistics School Beijing Wuzi University Beijing China

**Keywords:** glucose tolerance, lipid profile, mushroom

## Abstract

Obesity has become one of the most important health problems worldwide requiring urgent need for efficient control. *Pleurotus* *citrinopileatus* (*P. citrinopileatus*)—a type of edible mushroom with abundant bioactive molecules—is a promising source for achieving this goal. In the present study, we evaluated the anti‐obesity and hypolipidemic effect of *P. citrinopileatus* water extract (PWE) using a series of biochemical assays in randomized high‐fat diet‐induced obese (DIO) C57BL/6J mice, which were gavaged daily with low or high levels of PWE (400 or 800 mg/kg of body weight, respectively) in addition to high‐fat diet for 12 weeks. Results showed that PWE significantly reduced the weight gain, fat accumulation, and food intake of DIO mice within 12 weeks. PWE also decreased the serum triglycerides, cholesterol and low‐density lipoprotein, aspartate transaminase, nonesterified fatty acid, and creatinine, but increased high‐density lipoprotein. Additionally, PWE improved the glucose tolerance of mice fed with high fat. From above, we conclude that PWE has great potential as functional foods for management of obesity and/or associated metabolic disorders.

## 1 BACKGROUND

Obesity is one of the most important health problems worldwide, especially in developed countries (Novick, 2016; Talmor & Dunphy, 2015). Obesity increases the risk of developing numerous diseases such as hyperlipidemia, diabetes, atherosclerosis, liver damage, and cancers (Zhao & Castonguay, 2017). Additionally, obesity also increases economic burden of the government (Withrow & Alter, 2011). Up to now, effective cure for obesity is lacking. Potential strategies for therapeutic intervention of obesity development include altering neural signals in the brain to regulate appetite, restricting nutrient absorption in the gut and promoting fat oxidation in adipose tissues (Pilch & Bergenhem, 2006). Discovering compounds that are capable of achieving this goal is attracting, especially those extracted from natural herbs (Ramawat, Dass, & Mathur, 2009), of which mushroom is such a resourceful material for exploration (Badalyan & Singh, 2014).

In Asia, mushrooms have been acclaimed as tonic products for thousands of years. *Pleurotus citrinopileatus* (also known as golden mushroom) is an edible mushroom of the genus *Pleurotus* that is recommended as healthful food with high protein and fiber content but low level of lipids (Alam et al., 2008; Ghosh, Mitra, & Chakravarty, 2008; Rodrigues et al., 2015). Accumulating evidence shows that water extracts from *P. citrinopileatus* have many beneficial functions including antitumor activity (Zhang et al., 1994), immune‐enhancing ability (Minato, 2008), and antihyperglycemic properties (Rushita, Vijayakumar, Noorlidah, Abdulla, & Vikineswary, 2013; Zheng, Jin, & Shi, 2012). Interestingly, both ethanolic and water extracts of *P. citrinopileatus* exhibited antioxidant activities (Lee, Huang, Liang, & Mau, 2007) and antihyperlipidemic effect in rats (Hu, Liang, et al., 2006). In addition, we recently showed that the ethanolic extract of *P. citrinopileatus* also had good anti‐obesity effect (Chi et al., 2017).

Water‐soluble polysaccharides from mushrooms were frequently shown to be beneficial in health maintenance (Xu et al., 2010; Yashvant, Naraian, & Singh, 2012). A recent report showed that polysaccharides extracted from *Ganoderma lucidum*—another type of well‐known edible fungi—effectively reduced body weight in mice by modulating the composition of the gut microbiota (Chang et al., 2017). Compared with *G. lucidum*,* P. citrinopileatus* is more widely cultured and easier to grow. Therefore, we were attempting to investigate the anti‐obesity and hypolipidemic functions of the water extract from *P. citrinopileatus* (PWE) in high‐fat diet‐induced obese (DIO) mice.

## 2 METHODS

### 2.1 Materials and chemicals

*Pleurotus citrinopileatus* was obtained from Beijing Shengxin Oasis International Technology Development Co., Ltd and grown in the experimental field in Beijing Vocational College of Agriculture. Fresh fruiting bodies of *P. citrinopileatus* were dried at 40°C. Animal diets were prepared from Beijing HFK Bioscience Co., Ltd. The high‐fat diet contains 20% kcal of carbohydrate, 20% kcal of protein, and 60% kcal of fat (based on research diets D12492), while the normal diet contains 70% kcal of carbohydrate, 20% kcal of protein, and 10% kcal of fat (based on research diets D12450B). The PWE was prepared as follows. The dried fruit bodies of *P. citrinopileatus* were incubated in 90°C hot water for 3.5 hr, and the solution was then filtered with Whatman No. 1 filter paper. The filtrate was concentrated under reduced pressure in an evaporator. The precipitate was then collected and freeze‐dried to obtain the water extract. As reported previously, the PWE has high content of water‐soluble polysaccharides as well as phenol compounds (12.38 mg/g) (He et al., 2016; Lee et al., 2007).

### 2.2 Animal experiment

Male C57BL/6J mice (6 weeks old) with good health condition were purchased from Vital River Laboratories Inc. (Beijing, China). The animals were housed and maintained under standard laboratory conditions (adequate fresh air exchange, temperature 20–24°C, and relative humidity 40%–70%) in the specific pathogen‐free animal laboratory of the Supervision & Testing Center for GMOs Food Safety, Ministry of Agriculture (Beijing, China), with the license number SYXK (Beijing) 2015‐0045. A 12‐hr light/dark automatic cycle of artificial illumination was used. The mice (*N* = 24) were randomly divided into four groups, each containing six mice. The vehicle control group received a normal diet (20% calories were provided from fat). The diet‐induced obese (DIO) mice were fed with high‐fat diet (60% calories were provided from fat). The DIO mice were considered obese when the weight was 15% higher than that of vehicle control mice after 11 weeks. One group of DIO mice fed with distilled water together with high‐fat diet was marked as negative control group. The other two groups of DIO mice were gavaged daily in the morning with low or high levels of PWE (400 or 800 mg/kg of body weight, respectively) in addition to high‐fat diet for 12 weeks (marked as low‐dose group and high‐dose group). At the end of the 12‐week experiment, all mice were decapitated under anesthesia of ether. All mice had free access to fresh diet and drinking water during the experimental period. The protocol was approved by Animal Ethics Committee of China Agricultural University (Beijing, China).

### 2.3 Biochemical assay of blood

Blood samples were collected from the socket of the eyeball into heparinized tubes. The biochemistry of the blood was analyzed as previously reported (Chi et al., 2017). The blood biochemical indices including fasting blood triacylglycerol (TG), total cholesterol (CHO), low‐density lipoprotein cholesterol (LDL), high‐density lipoprotein cholesterol (HDL), alanine aminotransferase (ALT), aspartate aminotransferase (AST), alkaline phosphatase (ALP), lactic dehydrogenase (LDH), blood urea nitrogen (BUN), and creatinine (CREA) were determined using a biochemical analyzer (Hitachi 7020, Japan). These indices are common indicators of dyslipidemia, and liver and kidney damages. The blood glucose was measured using a blood glucose meter (Accu‐Chek Performa; Roche).

### 2.4 Statistical analysis

Data were presented as mean ± standard deviation. Single‐factor analysis of variance followed by a two‐tailed Student's *t* test was used for comparison using SPSS 20.0. The significance was set at *p* < 0.05.

## 3 RESULTS

### 3.1 PWE reduced weight gain in high‐fat diet‐fed mice

**Figure 1:**
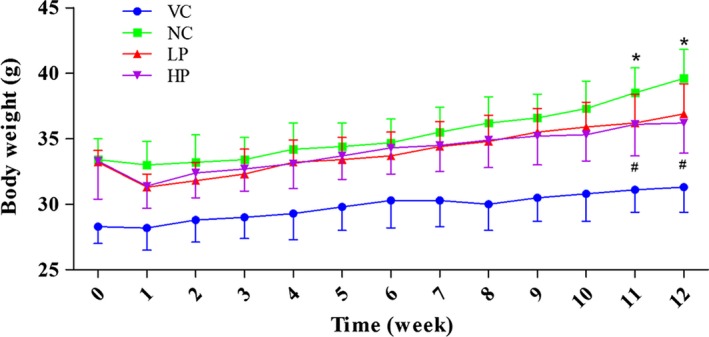
Effect of PWE on body weight in mice. The body weight of DIO mice was higher than that of the normal mice during 12 weeks. PWE slowed down the body weight gain in DIO mice fed with a high‐fat diet. Value = means ± *SD* (*n* = 6), * significant difference between LP and NC (*p* < 0.05); ^#^ denotes significant difference between HP and NC groups (*p* < 0.05). HP: high dose; LP: low dose; NC: negative control; VC: vehicle control

Throughout the 12 weeks’ treatment, the body weight of each mouse was recorded every week (Figure 1). At the beginning, the weight of DIO mice (33.6 g vs. 28.2 g/mouse on average) was 16.1% higher than that of the mice fed with normal diet, indicating that the DIO mice model was successfully established. In the negative control group, the mice had an average increase in body weight by nearly 18.6% over 12 weeks, whereas the weight increase was only 8.1% and 4.8% for low‐dose and high‐dose groups of mice, respectively (*p* < 0.05) (Figure 1).

### 3.2 PWE reduced food intake in DIO mice

Food intake of the mice in low‐dose and high‐dose groups (62 ± 4.6 g, 41 ± 4.2 g, respectively) was lower than that of mice in negative control group (94 ± 3.2 g). Food efficiency ratio of the mice in low‐dose (1.2%) and high‐dose groups (1.1%) was also lower than that in negative control group (2.5%) (Table 1).

**Table 1:** Effect of PWE on food intake (g) and food efficiency ratio in mice

Weeks	1	2	3	4	5	6	7	8	9	10	11	12	Food efficiency ratio (%)
VC	84 ± 4.2	81 ± 2.8	71 ± 14.3	101 ± 0.6	120 ± 0	88 ± 3.3	83 ± 3.9	92 ± 2	85 ± 5.4	95 ± 9.6	95 ± 9.6	92 ± 14.1	1.11
NC	79 ± 1.4	71 ± 9.9	67 ± 5.8	98 ± 7.4	66 ± 20.3	80 ± 0.3	83 ± 3.7	79 ± 11.2	82 ± 7.8	94 ± 3.2	94 ± 3.2	83 ± 8.8	2.54
LP	77 ± 6.4	81 ± 5.7	80 ± 2.8	85 ± 16.3	86 ± 6.7	73 ± 3.5	71 ± 4.2	69 ± 7.1	79 ± 4.2	64 ± 1.8	62 ± 4.6	75 ± 8.5	1.20
HP	77 ± 0.7	83 ± 4.9	81 ± 3.5	72 ± 31.1	86 ± 12.9	52 ± 17.2	46 ± 17.9	67 ± 4.2	83 ± 12	41 ± 4.2	41 ± 4.2	78 ± 8.5	1.14

*Notes*

Data are means ± *SD* (*n* = 2), and the values are from two cages with each cage containing three mice. Food efficiency ratio = body weight gain/food intake.

HP: high dose; LP: low dose; NC: negative control; VC: vehicle control.

### 3.3 PWE reduced fat accumulation

High‐fat diet significantly increased epididymal and groin fat mass (*p* < 0.05) (Table 2). The weight of epididymal fat relative to body weight was lower in low‐dose and high‐dose groups of mice than that in negative control group by 15.0% and 34.7% (*p* < 0.05), respectively. The weight of groin fat relative to body weight was also lower in low‐dose and high‐dose groups of mice than that of mice in negative control group by 14.4% and 42.1%, respectively (*p* < 0.05).

**Table 2:** Effect of PWE on body fat weight (g) and organ weight/body weight ratio in mice

Group	Epididymis adipose	Groin adipose	Heart	Lung	Liver	Kidney	Spleen
VC	0.95 ± 0.30	0.81 ± 0.14	0.59 ± 0.07	0.72 ± 0.11	4.32 ± 0.14	1.25 ± 0.17	0.28 ± 0.08
NC	4.32 ± 0.57*	2.92 ± 1.10*	0.47 ± 0.08	0.55 ± 0.08	3.60 ± 0.11*	1.18 ± 0.12	0.25 ± 0.06
LP	3.67 ± 1.09*	2.5 ± 1.28* ^,^ **	0.57 ± 0.11	0.56 ± 0.10	4.08 ± 0.58	1.29 ± 0.14**	0.27 ± 0.11
HP	2.82 ± 0.63* ^,^ **	1.69 ± 0.44* ^,^ **	0.56 ± 0.05**	0.6 ± 0.06*	3.83 ± 0.33*	1.28 ± 0.09	0.29 ± 0.05

*Notes*

Data are means ± *SD* (*n* = 6).

HP: high dose; LP: low dose; NC: negative control; VC: vehicle control.

^*^Denotes significant difference compared with VC (*p* < 0.05), ^**^Denotes significantly difference compared with NC (*p* < 0.05).

Generally, high fat increased organ weight relative to the body weight, while PWE alleviated this symptom. Typically, heart weight relative to body weight was significantly higher in high‐dose group of mice than that in negative control group (negative control group: 0.47% ± 0.08%, high‐dose group: 0.56% ± 0.05%) (*p* < 0.05). Liver weight to body weight ratio in low‐dose and high‐dose groups was increased but not significantly in low‐dose or high‐dose groups of mice (negative control group: 3.60 ± 0.11, low‐dose group: 4.08 ± 0.58, high‐dose group: 3.83 ± 0.33). Kidney weight relative to body weight was significantly increased in mice in low‐dose group compared to that in negative control group (negative control group: 1.18% ± 0.12%, low‐dose group: 1.29% ± 0.14%) (*p* < 0.05).

### 3.4 PWE improved lipid profile and other blood biochemistry indices

**Figure 2:**
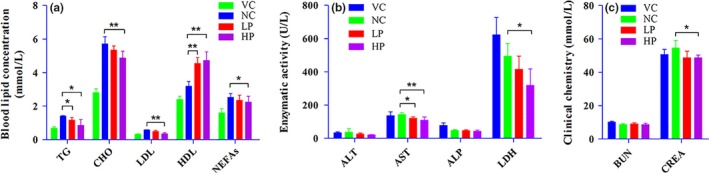
Effect of PWE on blood lipid profile, enzymatic activity, and clinical biochemistry in mice. (a) PWE slightly decreased the nonesterified fatty acids in mice fed with a high‐fat diet, but this difference failed to reach a significant level. (b) There was no significant difference in the levels of several enzymes (ALT, AST, ALP, and LDH) between the DIO mice and the VC mice. PWE decreased the AST at both low and high doses, whereas only a high dose of PWE decreased LDH in DIO mice. (c) A high dose of PWE significantly lowered the CREA content in mice. Value = means ± *SD* (*n* = 6), *p < 0.05, **p < 0.01, LP/HP compared with NC. ALP: alkaline phosphatase; ALT: alanine aminotransferase; AST: aspartate aminotransferase; BUN: blood urea nitrogen; CREA: creatinine; HP, high dose; LDH: lactic dehydrogenase; LP: low dose; NC: negative control; VC: vehicle control

The mice in negative control group had significantly higher serum total triglycerides (negative control group: 1.40 ± 0.04 mmol/L, vehicle control group: 0.69 ± 0.08 mmol/L), serum total cholesterol (negative control group: 5.71 ± 0.43 mmol/L, vehicle control group: 2.81 ± 0.22 mmol/L), and LDL (negative control group: 0.58 ± 0.02 mmol/L, vehicle control group: 0.33 ± 0.03 mmol/L) compared to those in vehicle control group (*p* < 0.01) (Figure 2a). PWE significantly reduced the blood triglyceride level after 12 weeks’ administration (negative control group: 1.40 ± 0.04 mmol/L, low‐dose group: 1.25 ± 0.11 mmol/L, high‐dose group: 1.06 ± 0.29 mmol/L, respectively) (*p* < 0.05) (Figure 2a). Thus, 400 and 800 mg/kg/day PWE treatment reduced the blood triglyceride levels by 10.9% and 24.4%, respectively, compared with the negative control mice. High dose of PWE significantly reduced cholesterol level (negative control group: 5.71 ± 0.43 mmol/L, high‐dose group: 4.12 ± 0.69 mmol/L) (*p* < 0.01) (Figure 2a). High dose of PWE also significantly reduced low‐density lipoprotein (negative control group: 0.58 ± 0.02 mmol/L, high‐dose group: 0.38 ± 0.02 mmol/L) (*p* < 0.01) and nonesterified fatty acids (negative control group: 1.60 ± 0.25 mmol/L, high‐dose group: 1.90 ± 0.65 mmol/L) (*p* < 0.05). Additionally, PWE efficiently increased high‐density lipoprotein (negative control group: 3.28 ± 0.28 mmol/L, low‐dose group: 4.63 ± 0.35 mmol/L, high‐dose group: 1.86 ± 0.42 mmol/L) (*p* < 0.05) (Figure 2a).

*Pleurotus citrinopileatus* water extract significantly reduced AST level (negative control group: 142.67 ± 11.36 U/L, low‐dose group: 114.00 ± 18.86U/L, high‐dose group: 96.83 ± 3.31U/L) (*p* < 0.01) (Figure 2b). High dose of PWE also significantly reduced CREA significantly (negative control group: 50.67 ± 3.01 mmol/L, HP: 47.17 ± 4.22 mmol/L) (Figure 2c) (*p* < 0.05).

### 3.5 PWE improved blood glucose condition

**Figure 3:**
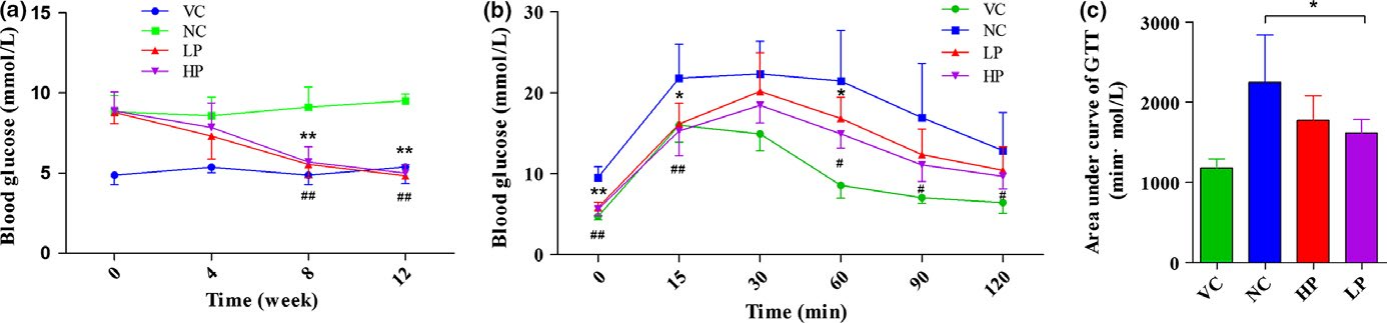
Effects of PWE on serum concentration of glucose and GTT in mice. (a) The DIO mice had a significantly higher glucose level compared with VC. Low dose or high dose of PWE reduced the blood glucose level to nearly normal. (b) The glucose concentration of DIO mice was significantly higher than that of the normal mice. PWE at either low or high doses significantly reduced glucose levels to nearly normal after 8 weeks. (c) High‐fat diet‐induced glucose intolerance in mice, while PWE reversed this condition. Area under curve was referred to the area under the curve of the glucose level from 0 to 120 min postinjection. There was a significant difference in glucose level and GTT between the VC mice and the NC mice, whereas no significant difference was found between LP mice and HP mice. Value = means ± *SD* (*n* = 6), * *p* < 0.05, ** *p* < 0.01 between LP and NC groups, # *p* < 0.05, ## *p* < 0.01 between HP and NC groups. HP: high dose; LP: low dose; NC: negative control; VC: vehicle control

Fasting serum glucose level in negative control group was higher than that in vehicle control group throughout 12 weeks (Figure 3a). The administration of PWE resulted in significant reduction of fasting blood glucose of mice starting from the 8th week (*p* < 0.01). Blood glucose concentrations were markedly elevated after glucose loading. Reduction in blood glucose concentration tended to be delayed in mice of negative control group compared to that in vehicle control group. Compared to the mice in negative control group, administration of high dose and low dose of PWE decreased the glucose response at 15 min by 26% and 30%, respectively (*p* < 0.05) (Figure 3b).

## 4 DISCUSSION

Obesity is associated with higher risk of many diseases such as type 2 diabetes, hyperlipidemia, and cardiovascular disease (Sullivan, Ghushchyan, & Ben‐Joseph, 2008). Therefore, the quest for effective strategies to prevent obesity has been intensified. Mushrooms with rich bioactive molecules have become attractive as a natural functional food (Meng et al., 2011). Several certain compounds or crude extracts from different types of mushrooms that can alleviate obesity and/or associated symptoms have been reported such as mushroom chitosan (Neyrinck et al., 2009). The current study tested *P. citrinopileatus*—an edible and easy‐to‐grow mushroom.

*Pleurotus citrinopileatus* water extract has many medicinal properties including antitumor functions (Zhang et al., 1994), immune modulating activity (Minato, 2008; Minato, Laan, Ohara, & Die, 2016), antihyperglycemic function, and blood‐lipid‐lowering effect (Hu, Wang, Lien, Liaw, & Lee, 2006). Consumption of high‐fat diet can result in impaired pancreatic function of insulin secretion, leading to glucose intolerance. PWE improved the condition of the DIO mice in glucose intolerance, which is a prediabetic state of hyperglycemia that is closely associated with obesity or metabolic syndrome (Huang, Chiang, Yao, & Chiang, 2010; Zhao & Castonguay, 2017). We further found that PWE treatment reduced body weight gain in DIO mice resulting from low food intake and food efficiency ratio as well as inhibition of adipogenesis. Therefore, PWE inhibited energy intake of mice and thus slowed down the positive energy balance with aging.

The anti‐obesity function of PWE was not caused by any toxic effect evidenced by the organ indices and serum biochemical factors. We did not find any apparent pathological change in any organ examined of the mice. Although some DIO mice showed reduced organ indices of heart, lung, liver, and kidney relative to the whole‐body weight, this was likely because of increased body weight instead of dysplasia. A high‐fat diet did not cause any significant change in several enzymes including ALT, AST, ALP, and LDH. On the contrary, PWE decreased the activity of AST and LDH as well as the CREA content indicating its potentially positive effect on liver and kidney functions.

Oral administration of PWE improved lipid profile of DIO mice in a dose‐dependent way, suggesting that PWE had therapeutic potential to prevent diet‐induced hyperlipidemia. This also explained a previous report that 5% *P. citrinopileatus* diet decreased the atherogenic lipid profile in the hypercholesterolemic rats (Alam, Yoon, Lee, Lee, & Lee, 2011). In addition, we found the blood glucose condition including the fasting glucose level and glucose tolerance were all improved by PWE in DIO mice. This was consistent with the previous research that water‐soluble polysaccharides extracted from fermented *P. citrinopileatus* alleviated the diabetic symptoms of diabetic rats as well as improved lipid profile (Hu, Wang, et al., 2006).

## 5 CONCLUSIONS

Our research firstly provided evidence that the PWE had potential in the prevention of obesity and prediabetes in mice. As obesity is usually associated with compromised oxidative condition, dyslipidemia (Franssen, Monajemi, Stroes, & Kastelein, 2011; Higdon & Frei, 2003; Kose et al., 2014), and deranged immune system (de Heredia, Gómez‐Martínez, & Marcos, 2012), its anti‐obesity effect is likely to be attributable to its ability of enhancing the activities of antioxidant enzymes, promoting the expression of their isozymes (Liu, Tao, Cheng, & Zhou, 2011), and modulating the immune functions (Minato et al., 2016). However, future study is required to illuminate its anti‐obesity mechanism and further characterize the fractions in PWE.

## CONFLICT OF INTEREST

The authors declare that they do not have any conflict of interest.

## ETHICAL REVIEW

This study was approved by the Institutional Review Board of China Agricultural University.
